# Exercise interventions on body composition and quality of life of overweight/obese breast cancer survivors: a meta-analysis

**DOI:** 10.1186/s12905-023-02627-2

**Published:** 2023-09-12

**Authors:** Hongchang Yang, Li Liu, Xiaoxia Zhang

**Affiliations:** 1 Physical Education Department, Hohai University, Nanjing, Jiangsu China; 2 Department of Rehabilitation, Brain Hospital Affiliated to Nanjing Medical University, No.264, Guangzhou Road, Gulou District, Nanjing, Jiangsu China; 3 Department of Kinesiology, Centenary College of Louisiana, 2911 Centenary Blvd, Shreveport, LA USA

**Keywords:** Breast cancer survivor, Body composition, Exercise intervention, Obesity, Quality of life

## Abstract

**Background:**

This meta-analysis aimed to assess the effects of exercise interventions on body composition and quality of life in overweight/obese breast cancer survivors.

**Methods:**

Eligible randomized controlled trials (RCTs) were searched from the Cochrane Library, PubMed, and Embase databases and assessed using the Cochrane Collaboration’s assessing risk tool. The effect size was pooled as weighted mean difference (WMD) for body composition variables (i.e., body mass index [BMI], body fat, body weight, fat mass, lean mass, bone mineral density) and quality of life (i.e., physical health and mental health), and the confidence interval (CI) was set as 95%. Since heterogeneity existed, subgroup analysis was conducted to detect the source of heterogeneity.

**Results:**

Eight articles from six RCTs containing 548 overweight/obese breast cancer survivors (BMI ≥ 25 kg/m^2^) were included and analyzed. Compared to routine care, exercise intervention significantly decreased the body mass index [WMD (95% CI) = -1.37 (-2.50, -0.23) kg/m^2^] and body fat [WMD (95% CI) = -3.80 (-6.59, -1.01) %] of overweight/obese breast cancer survivors. Exercise intervention showed a tendency to increase physical health [WMD (95% CI) = 2.65 (-10.19, 15.48)] and mental health [WMD (95%CI) = 1.38 (-4.18, 6.95)], but no statistical significance was observed. A subgroup analysis showed the duration of intervention was a source of heterogeneity on body composition. In the 16-week subgroup, exercise intervention decreased fat mass and BMI while increased lean mass and bone mineral density. The 52-week exercise intervention was effective in increasing lean mass. A significant exercise intervention effect on reducing body fat was only detected in the 12-week subgroup.

**Conclusion:**

Exercise intervention significantly decreased the body mass index and body fat of overweight/obese breast cancer survivors. The benefits of exercise interventions for overweight/obese breast cancer survivors need more evidence from high-quality RCTs with large sample sizes.

**Supplementary Information:**

The online version contains supplementary material available at 10.1186/s12905-023-02627-2.

## Background

Breast cancer is the most common malignancy in women [1], which is the fifth leading cause of cancer-related deaths with approximately 6.9% of them being reported worldwide in 2020 [2]. With advances in early screening and breast cancer treatments, breast cancer mortality has markedly declined during the last couple of decades, which has resulted in an increasing number of breast cancer survivors [3]. There was a 39% decrease in breast cancer mortality in the United States from 1989 to 2015, translating to approximately 322,600 patients saved from breast cancer-related deaths [4]. Despite the increasing survival rate, many breast cancer survivors frequently encounter chronic complications or long-term treatment sequelae that significantly impact their quality of life. These complications may include pain, limited upper limb function, fatigue, lipid disorders, obesity, premature menopause, and lymphedema [5–7]. It is crucial of secondary prevention or intervention strategies to maintain the overall health of the breast cancer survivors.

Exercise has traditionally not been advised in patients with cancer [8]. However, in recent years, studies on exercise have revealed the potential benefits for cancer survivors [9, 10]. Exercise is one type of physical activity that is planned, structured, and repetitive with an objective to improve or maintain physical fitness (such as cardiorespiratory endurance, muscular endurance, muscular strength, body composition, and flexibility) [11]. The American College of Sports Medicine roundtable proposes that exercise is safe for cancer survivors and advocates that they avoid inactivity, which helps to improve physical functioning, anxiety and depressive symptoms, cancer-related fatigue, and quality of life [8, 12]. Numerous studies have provided evidence for the benefits of exercise for breast cancer survivors, including improved shoulder range of motion and muscular strength, reduced anxiety and cancer-related fatigue, and enhanced self-esteem and overall well-being [13–16]. For instance, structured exercise programs have been shown to enhance shoulder range of motion in postoperative breast cancer patients [13]. Aerobic exercise has also been found to be effective in reducing cancer-related fatigue (CRF) among breast cancer survivors [14]. Overall, these findings provide compelling evidence for the benefits of incorporating exercise interventions in the care of breast cancer survivors.

However, it remains unclear the effects of exercise on body composition and quality of life among overweight or obese breast cancer survivors. Approximately 50% of breast cancer survivors worldwide are overweight or obese, and they usually gain more body weight following hormonal or adjuvant therapy [15]. According to the weight status classification established by World Health Organization and the National Institutes of Health individuals with a body mass index (BMI) of 30 kg/m^2^ or above are classified as obese and those with a BMI ranging from 25.0 to 29.9 kg/m^2^ are considered overweight [16, 17]. Breast cancer survivors are more likely to be obese, which is generally related to worse health-related quality of life [18]. In addition, overweight/obese breast cancer survivors have been documented to have a high risk of recurrence, all-cause mortality, and long-term morbidities (e.g., cardiovascular disease, diabetes) [19–21]. Studies have indicated that exercise and weight loss interventions (incorporating diet, exercise and psychosocial support) may improve the quality of life and reduce BMI, body weight and waist circumference of breast cancer survivors [22, 23]. While some other studies reported limited effect of exercise on body composition, such as body weight [24, 25], BMI [26, 27], fat, and lean mass [25, 28], or health-related quality of life [29, 30] among obese/overweight breast cancer survivors.

Previous research has shown that compared to normal-weight women, overweight or obese women had different energy expenditures in a day (higher portions in sedentary and light physical activity) [31]; when completing same intensity exercise, overweight or obese women utilized significantly more calories than normal-weight women [32]. These studies suggest overweight or obese women may experience different effects from exercise. Although exercise interventions had been proved to have a significant impact on improving health-related quality of life and reducing body weight and waist circumference in breast cancer survivors [33], no consensus has been made among overweight or obese breast cancer survivors . Therefore, we conducted a meta-analysis based on randomized controlled trials (RCTs) to assess the effects of exercise interventions on the body composition and quality of life of overweight/obese breast cancer survivors.

## Materials and methods

### Screening eligible studies

Studies that met all the following criteria were included: (1) involving breast cancer survivors who were overweight or obese; (2) reporting the differences in outcomes between the exercise and control groups (routine care); (3) involving one or more of the following outcomes, including body weight, BMI, fat mass, lean mass, bone mineral density, body fat, and 36-Item Medical Outcomes Survey-Short Form (SF-36); and (4) studies that were RCT. The exclusion criteria were (1) reviews, conference abstracts, comments, and other non-original articles; (2) involving other strategies in addition to exercise interventions with weight loss as the primary goal; and (3)involving participants who had regular daily exercise habits. If any of the exclusion criteria was met, the study will not be included in the meta-analysis. Additionally, if multiple studies utilized the same dataset, only the study with the most detailed data will be extracted and analyzed.

### Retrieval strategies

To investigate the effect of exercise on body composition and quality of life in overweight/obese breast cancer survivors, studies were retrieved from PubMed, Cochrane Library, and Embase databases until June 9, 2022, without language restrictions. The retrieval terms were as follows: “overweight” OR “obese” OR “obesity” AND “exercise” OR “physical activity” OR “sports” OR “training” OR “exercising” AND “breast neoplasms” OR “breast cancer.” Additionally, references cited in the included studies and relevant reviews were retrieved.

**Fig. 1:**
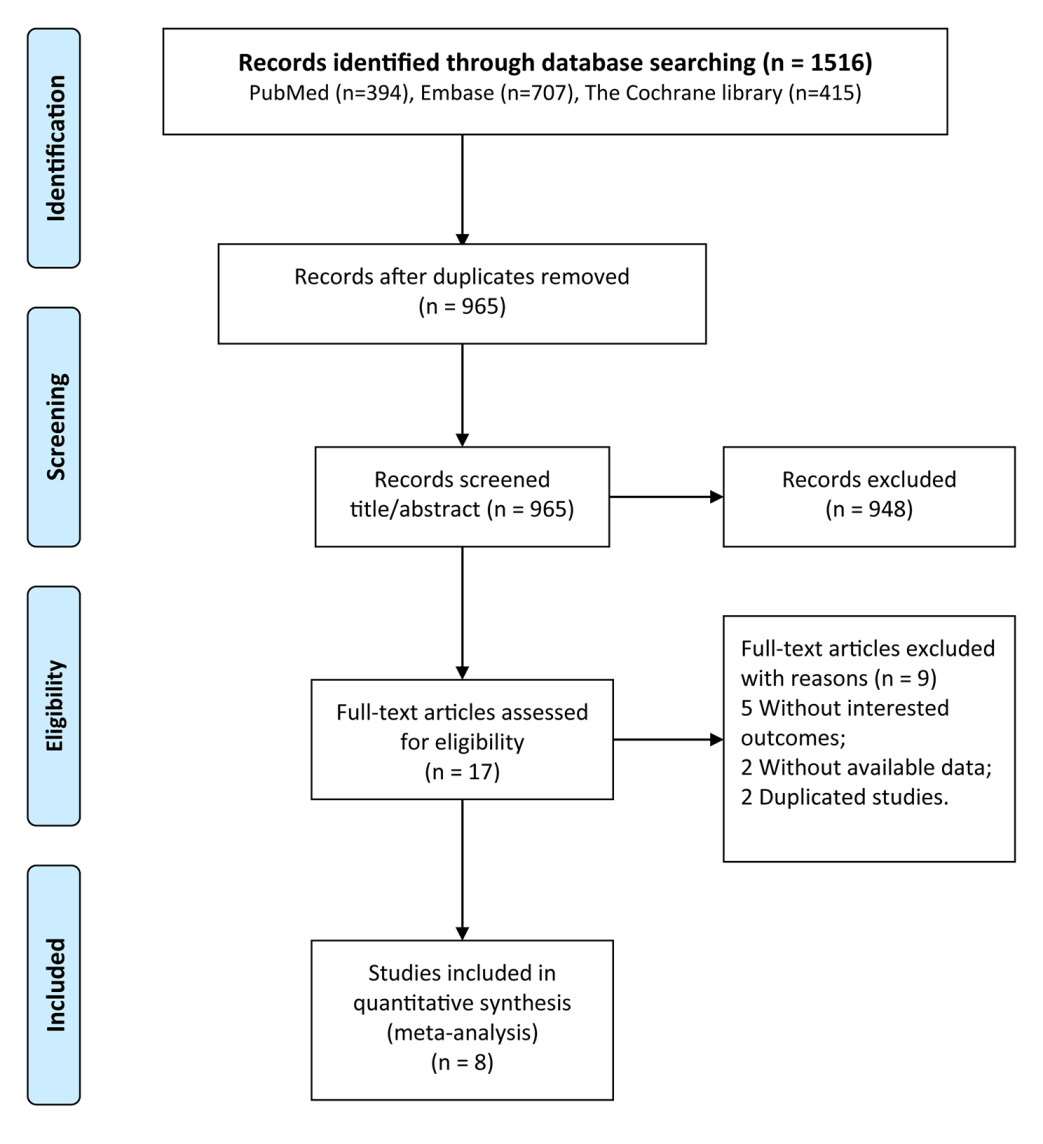
Processes of study selection

### Outcome variables

**Fig. 2:**
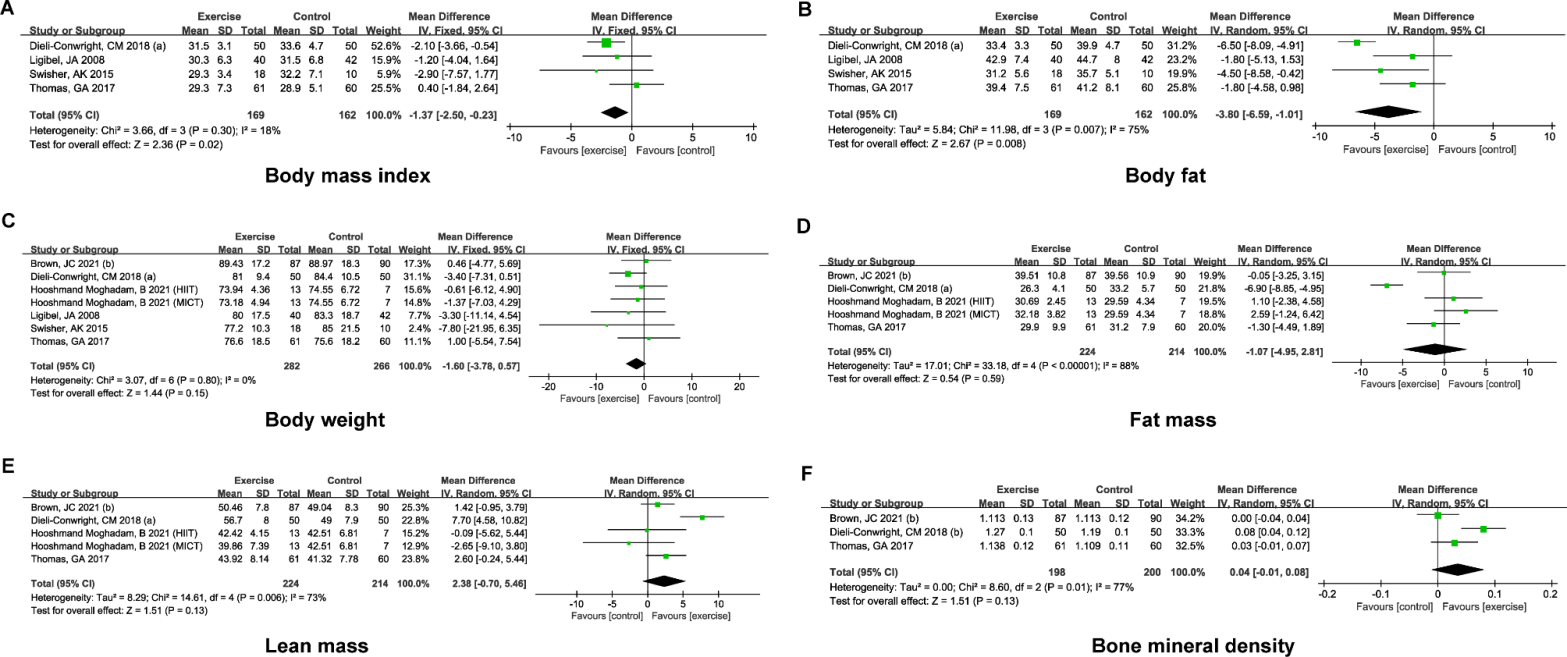
Effect of exercise intervention on body composition

**Fig. 3:**
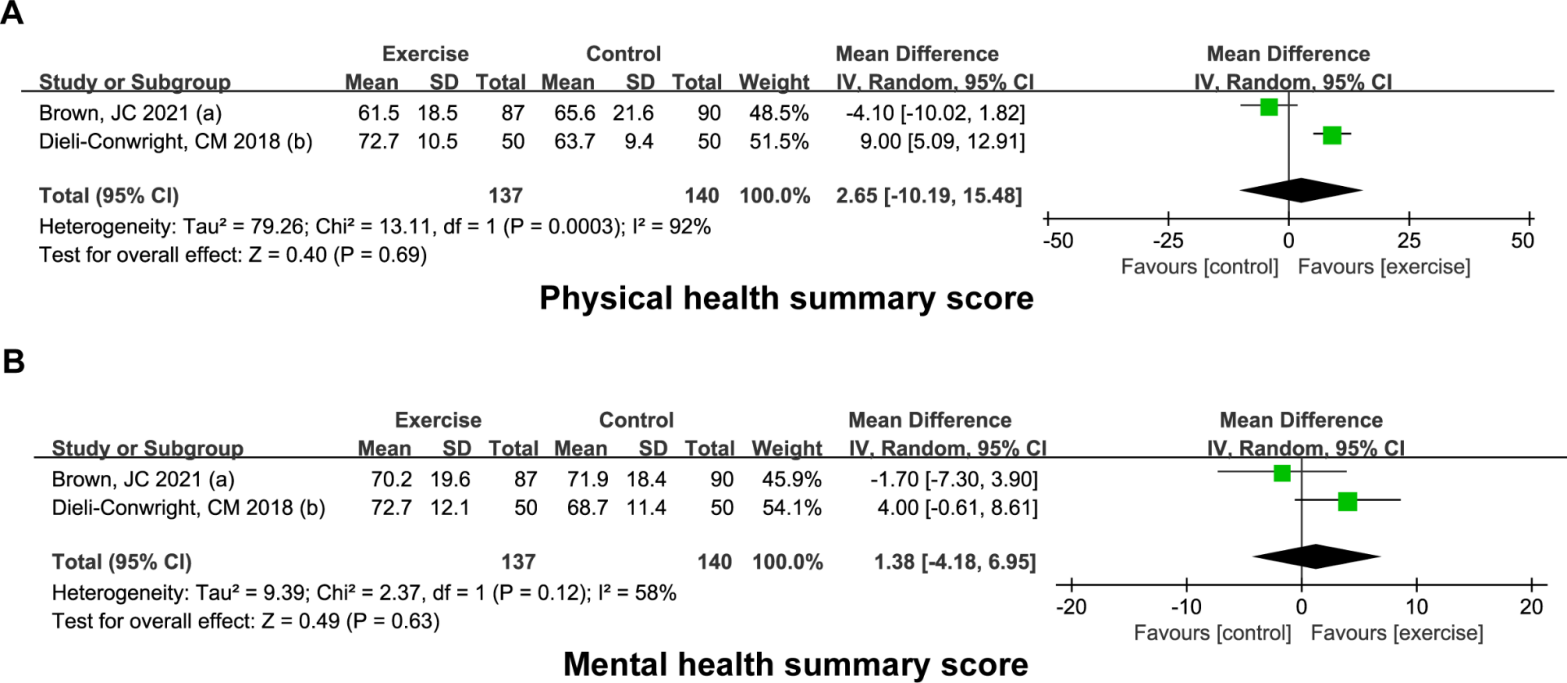
Effect of exercise intervention on quality of life Forest plots showing the pooled results of the exercise intervention on the physical health summary score **(A)** and mental health summary score **(B)** of breast cancer survivors

This study examined several outcome variables, including body composition indicators (BMI and body weight, fat mass, lean mass, and bone mineral density) and quality of life (physical and mental health summary score). Body composition indicators were assessed using internationally standardized metrics, while quality of life was assessed using validated scales. All outcome variables were treated as continuous data in the analysis (see details in Figs. 2 and 3).

### Extracting data and quality assessment

The following information was extracted: name of the first author, year of publication, area in which the study was conducted, intensity of exercise of the involved participants, definition of overweight and obesity, exercise intervention program, demographic information of the involved participants (age, and race), and outcomes (BMI, body weight, fat mass, lean mass, bone mineral density, quality of life). Information extraction was completed by two independent investigators, and the third researcher would be consulted for resolution in case of any disagreements. The Cochrane Collaboration tool was used to assess the risk of bias in the included RCT studies, which encompasses six domains of bias, namely selection bias, performance bias, detection bias, attrition bias, reporting bias, and other bias. The included randomized controlled trials (RCTs) were categorized into low risk,or unclear risk in the aforementioned domains [34].

### Statistical analysis

All statistical analyses were performed using RevMan 5.3 and Stata12.0. The weighted mean difference (WMD) and 95% confidence interval (CI) were used as effect sizes to pool the results. WMD is a statistical measure used to assess the difference in continuous variables between two groups, which calculates the average difference between the two groups, with each study’s contribution weighted by the sample size. A narrower 95% CI indicates a more precise estimate. Cochran’s Q test and the I^2^ test were used to assess heterogeneity of all outcome variables. A random-effects model was used for the meta-analysis when there was significant heterogeneity (*P* < 0.05, I^2^ > 50%); otherwise, a fixed-effects model was used (*P* ≥ 0.05, I^2^ ≤ 50%). The random-effects model allows for the possibility that studies are drawn from different populations, which means that differences across studies may be due to unidentified sources of variation. This model provides a more conservative estimate of treatment effects by considering both the within-study sampling error and the between-study variability [35]. A subgroup analysis was conducted in groups divided by the duration of the intervention. Sensitivity analysis was performed using a one-study-removed approach to assess the significant impact of individual included studies on the results of the meta-analysis [36]. Egger’s test [37] and funnel plot [38] was used to assess publication bias among the studies.

## Results

### Screening eligible studies

The workflow of the study retrieval and screening is shown in Fig. 1. First, we retrieved 1,516 studies from three databases and excluded 551 repetitive studies. Among the remaining 965 studies, 948 that did not meet the inclusion criteria were removed after reviewing the title and abstract. Nine studies were excluded after reviewing their full text. Finally, we identified eight eligible studies [23–30] for this meta-analysis.

### Detailed information for the included studies

A total of 8 articles were assessed and found to meet the inclusion criteria. Among them, two articles [23, 29] from the same RCT by Brown et al. (NCT01515124) and two articles [24, 30] from the same RCT by Dieli-Conwright et al.(NCT01140282) met the inclusion criteria. A total of 548 participants were included, ranging from 28 to 177 participants in different studies. All participants were breast cancer survivors with stages I-III tumors and with BMI equal or greater than 25 kg/m^2^. The study by Hooshmand et al. [25] was conducted in Iran, and the other studies were all conducted in the United States. Heterogeneity was observed among the studies on the intensity of exercise and exercise intervention programs of the participants (Table 1). The publication bias analysis resulted a moderate methodological quality of the included studies (See details in Supplementary Fig. 1).

**Table 1:** Characteristics of eight included studies in this meta-analysis

Study	PA, weekly, baseline	Definition of OW or obese	Exercise intervention	Intervention time, weeks	Group	n	Age, years	BMI, kg/m^2^	Race, White/ Black/Other
Brown, JC 2021 (a)	Not engaging in any RE or ≥ 3 bouts of AE of moderate intensity	BMI 25–50	Moderate-intensity AE was prescribed to a goal of 180 min weekly distributed over 3–6 days per week	52	Exercise	87	59.1 (8.1)	34.0 (6.2)	50/36/1
Brown, JC 2021 (b)				52	Control	90	59.0 (8.5)	34.0 (5.7)	66/22/2
Dieli-Conwright, CM 2018 (a)	Less than 60 min of structured exercise	BMI ≥ 25 kg/m^2^; BFP > 30%; WC > 88 cm	150 min of AE and 2–3 days of RE weekly	16	Exercise	50	52.8 (10.6)	33.1 (5.7)	39/3/8
Dieli-Conwright, CM 2018 (b)				16	Control	50	53.6 (10.1)	33.4 (5.2)	42/1/7
Hooshmand Moghadam, B 2021	Less than 60 min of physical activity	BMI ≥ 25 kg/m^2^; BFP > 30%	Supervised exercise on a cycle ergometer three days/week		HIIT	13	50–75	28.2 (2.2)	NR
				12	MICT	13	50–75	28.2 (2.2)	NR
					Control	14	50–75	28.2 (2.2)	NR
Ligibel, JA 2008	Less than 40 min per week	BMI ≥ 25.0 kg/m^2^; BFP > 30%	Two supervised 50-minute RE per week; 90 min of home-based AE weekly	16	Exercise	40	52 (9)	NR	NR
					Control	42	53 (9)	NR	NR
Swisher, AK 2015	NR	BMI ≥ 25 kg/m^2^	Supervised, moderate-intensity AE three times and 2 unsupervised sessions/week	12	Exercise	18	53.8 (43–65)	30.9 (3.3)	NR
					Control	10	53.6 (36–71)	32.5 (7.1)	NR
Thomas, GA 2017	Less than 90 min of physical activity	BMI ≥ 25 kg/m^2^	Twice-weekly supervised RE, 150 min of moderate-intensity AE	52	Exercise	61	62.0 (7.0)	30.0 (6.8)	53/7/1
					Control	60	60.5 (7.0)	28.7 (5.5)	54/4/2

AE, aerobic exercise; BFP, body fat percentage; BMI, body mass index; HIIT, high intensity interval training; MICT, moderate-intensity continuous training; NR, not reported; OW, overweight; PA, physical activity; RE, resistance exercise; WC, waist circumference

### Effect of exercise intervention on body composition

Four studies reported differences in the BMI of overweight/obese breast cancer survivors between the exercise and control groups, and there was no significant heterogeneity (I^2^ = 18%, *P* = 0.30). The forest plot revealed that the exercise intervention markedly decreased the BMI [WMD (95% CI) = -1.37 (-2.50, -0.23) kg/m^2^, *P* = 0.02] in overweight/obese breast cancer survivors (Fig. 2A). There was significant heterogeneity among the four studies reporting body fat (I^2^ = 75%, *P* = 0.007), and the pooled results indicated that exercise intervention markedly decreased body fat [WMD (95% CI) = -3.80 (-6.59, -1.01) %, *P* = 0.008] in overweight/obese breast cancer survivors (Fig. 2B). Nevertheless, the exercise intervention did not exert a significant effect on the body weight [WMD (95% CI) = -1.60 (-3.78, 0.57) kg, *P* = 0.15], fat mass [WMD (95% CI) = -1.07 (-4.95, 2.81) kg, *P* = 0.59], lean mass [WMD (95% CI) = 2.38 (-0.70, 5.46) kg, *P* = 0.13], and bone mineral density [WMD (95% CI) = 0.04 (-0.01, 0.08) g/m^2^, P = 0.13] of overweight/obese breast cancer survivors. The heterogeneity of the included studies was as follows: body weight (I^2^ = 0%, P = 0.8), fat mass (I^2^ = 88%, P＜0.00001), lean mass (I^2^ = 73%, P = 0.006), and bone mineral density (I^2^ = 77%, P = 0.01) (Fig. 2C-F). In summary, in comparison to routine care, exercise intervention significantly decreased the BMI and body fat of overweight/obese breast cancer survivors but had no significant influence on body weight, fat mass, lean mass, and bone mineral density.

Forest plots showing the pooled results of exercise intervention on BMI (A), body fat (B), body weight (C), fat mass (D), lean mass (E), and bone mineral density (F) of overweight/obese breast cancer survivors.

### Effect of exercise intervention on quality of life

There were only two studies reporting the differences in the physical health summary score (PHS) or the mental health summary score (MHS) of overweight/obese breast cancer survivors between exercise and control groups, and there was significant heterogeneity (I^2^ = 92%, *P* = 0.0003 for PHS; I^2^ = 58%, *P* = 0.12 for MHS). Forest plots revealed that exercise intervention had no significant influence on PHS [WMD (95% CI) = 2.65 (-10.19, 15.48), *P* = 0.69] and MHS [WMD (95% CI) = 1.38 (-4.18, 6.95), *P* = 0.63] in overweight/obese breast cancer survivors (Fig. 3).

### Subgroup analysis

We conducted analyses for all covariates and observed significant heterogeneity among the remaining covariates, except for exercise duration. Consequently, it was deemed inappropriate to convert them into grouping variables for subgroup analysis. Therefore, we solely performed subgroup analysis on exercise duration. Table 2 shows the results of the subgroup analyses based on the duration of intervention (12 weeks, 16 weeks, and 52 weeks). There was significant heterogeneity among the studies for BMI, body fat, fat mass, lean mass, and bone mineral density, and the heterogeneity test in each subgroup suggested that intervention duration was one of the sources of heterogeneity (Table 2). Specifically, in the 16-week subgroup, exercise intervention decreased fat mass [WMD (95% CI) = -6.90 (-8.85, -4.95) kg, *P* < 0.001], while increased lean mass [WMD (95% CI) = 7.70 (4.58, 10.82) kg, *P* < 0.001], and bone mineral density [WMD (95% CI) = 0.08 (0.04, 0.12) g/m^2^, *P* < 0.001] of overweight/obese breast cancer survivors (Table 2). Long-term exercise intervention (52 weeks) also revealed to be effective in increasing the lean mass [WMD (95% CI) = 1.91 (0.09, 3.73) kg, *P* = 0.04]. The subgroup analyses revealed significant exercise intervention effect on BMI in the 16-week subgroup [WMD (95% CI) = -1.89 (-3.26, -0.52) kg/m^2^, *P* = 0.007], but there were no significant effects on BMI at 12-week [WMD (95% CI) = 0.40 (-1.84, 2.64) kg/m^2^, *P* = 0.726] and 52-week groups [WMD (95% CI) = -2.90 (-7.57, 1.77) kg/m^2^, *P* = 0.224]. A significant exercise intervention effect on body fat was detected in the 12-week subgroup [WMD (95% CI) = -4.50 (-8.59, -0.42) %, P = 0.031] but not in the 16-week [WMD (95% CI) = -4.39 (-8.97, 0.19) %, P = 0.061] and 52-week groups [WMD (95% CI) = -1.80 (-4.58, 0.98) %, P = 0.205]. There was only one study in the 12- and 52-week subgroups for BMI and body fat, respectively (Table 2). Only two studies examined PHS and MHS as outcomes; therefore, subgroup analysis was not performed for these two outcomes.

**Table 2:** Results of subgroup analyses

Outcomes	No. of study	WMD (95%CI)	*P* value	Heterogeneity test	
				I^2^ (%)	P_H_
Body weight, kg	7	-1.60 (-3.78, 0.57)	0.15	0	0.80
Intervention time, weeks					
52	2	0.67 (-3.41, 4.76)	0.75	0	0.90
16	2	-3.38 (-6.88, 0.12)	0.06	0	0.98
12	3	-1.47 (-5.28, 2.33)	0.45	0	0.65
Fat mass, kg	5	-1.07 (-4.95, 2.81)	0.59	88	< 0.00001
Intervention time, weeks					
52	2	-0.68 (-2.93, 1.58)	0.56	0	0.59
16	1	-6.90 (-8.85, -4.95)	< 0.001	NA	NA
12	2	1.77 (-0.80, 4.35)	0.18	0	0.57
Lean mass, kg	5	2.38 (-0.70, 5.46)	0.13	73	0.006
Intervention time, weeks					
52	2	1.91 (0.09, 3.73)	0.04	0	0.53
16	1	7.70 (4.58, 10.82)	< 0.001	NA	NA
12	2	-1.17 (-5.37, 3.02)	0.583	0	0.56
BMI, kg/m^2^	4	-1.37 (-2.50, -0.23)	0.02	18	0.30
Intervention time, weeks					
52	1	0.40 (-1.84, 2.64)	0.726	NA	NA
16	2	-1.89 (-3.26, -0.52)	0.007	0	0.59
12	1	-2.90 (-7.57, 1.77)	0.224	NA	NA
Body fat, %	4	-3.80 (-6.59, -1.01)	0.008	75	0.007
Intervention time, weeks					
52	1	-1.80 (-4.58, 0.98)	0.205	NA	NA
16	2	-4.39 (-8.97, 0.19)	0.061	87	0.01
12	1	-4.50 (-8.59, -0.42)	0.031	NA	NA
BMD, g/m^2^	3	0.04 (-0.01, 0.08)	0.13	77	0.01
Intervention time, weeks					
52	2	0.01 (-0.02, 0.04)	0.365	6	0.30
16	1	0.08 (0.04, 0.12)	< 0.001	NA	NA

NA, not available; BMI, body mass index; BMD, bone mineral density

### Sensitivity analysis and publication bias

**Fig. 4:**
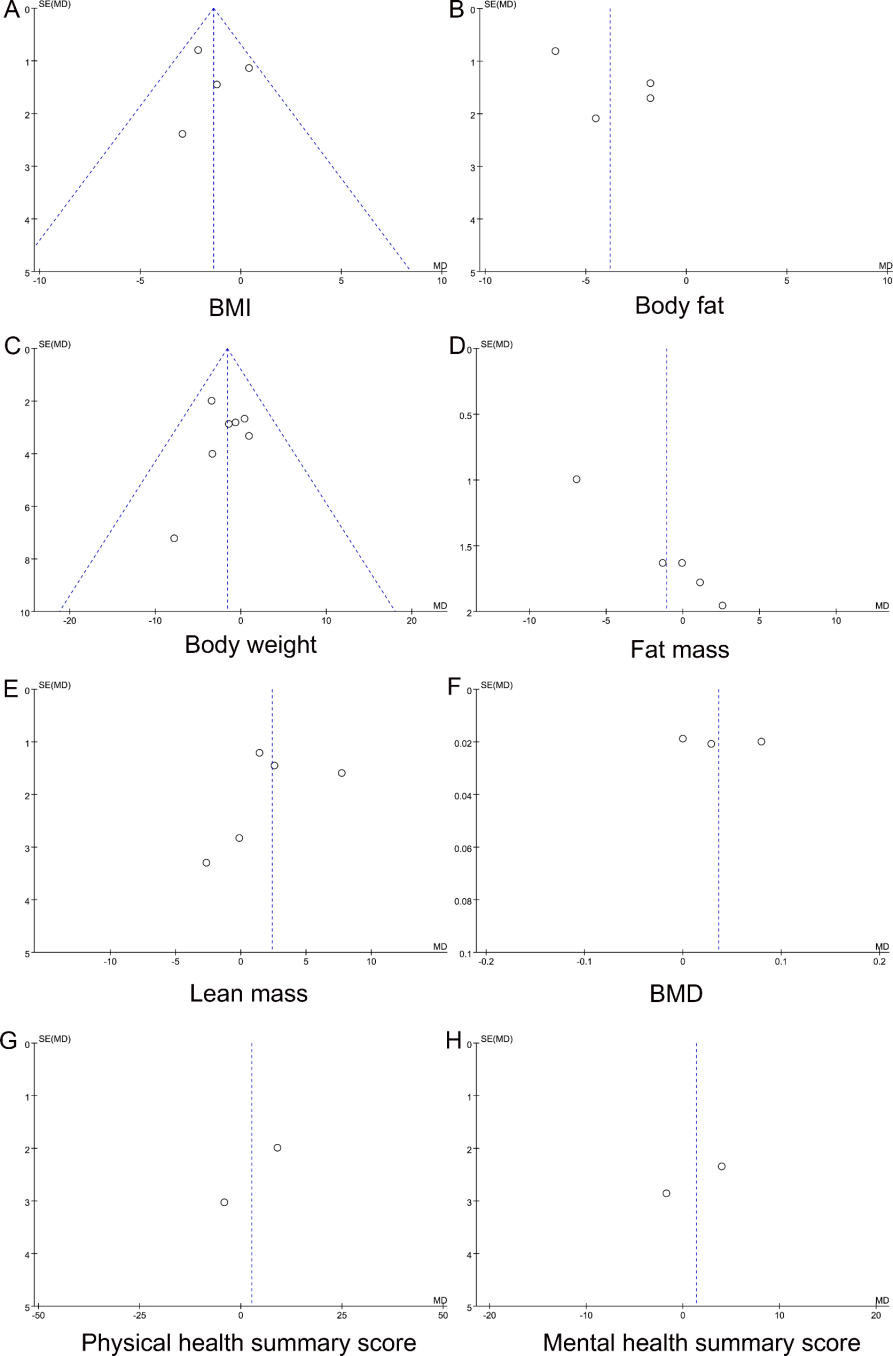
Funnel plot of body composition and quality of life

In sensitivity analysis, the pooled results for BMI changed from significant [WMD (95% CI) = -1.97 (-3.28, -0.66) kg/m^2^] to non-significant [WMD (95% CI) = -0.55 (-2.19, 1.10) kg/m^2^] after eliminating studies one by one, indicating an unstable result. For the other five outcomes, sensitivity analysis revealed stable results (Table 3). In addition, no publication bias was detected among the studies for any of the six outcomes (Table 3). Moreover, the funnel plot and scatter distribution of each outcome measure appear to be relatively symmetrical, indicating the absence of publication bias (Fig. 4A-H). There were only two studies on PHS and MHS; therefore, sensitivity analysis and publication bias tests were not performed for these two outcomes.

**Table 3:** Outcomes of the sensitivity analysis and test of publication bias

Outcomes	No. of studies	Sensitivity analysis		Egger’ s test
		WMD (95% CI)	Robust	*P* value
Body weight	7	-2.04 (-4.43, 0.36) to -1.64 (-4.00, 0.72)	Yes	0.768
Fat mass	5	-1.93 (-6.06, 2.20) to 0.39 (-1.31, 2.09)	Yes	0.051
Lean mass	5	1.42 (-0.25, 3.09) to 3.13 (-0.04, 6.31)	Yes	0.595
BMI	4	-1.97 (-3.28, -0.66) to -0.55 (-2.19, 1.10)	No	0.932
Body fat	4	-4.53 (-7.57, -1.49) to -3.56 (-7.12, -0.01)	Yes	0.232
BMD	3	0.01 (-0.02, 0.04) to 0.055 (0.005, 0.105)	Yes	0.688

BMI, body mass index; BMD, bone mineral density

## Discussion

This meta-analysis presented several important findings regarding the exercise effects on body composition and quality of life among overweight/obese breast cancer survivors. Compared to routine care, exercise intervention significantly decreased the BMI and body fat of overweight/obese breast cancer survivors. Meanwhile, exercise intervention decreased fat mass and increased lean mass and bone mineral density in overweight/obese breast cancer survivors while only under certain intervention periods (i.e., 16 weeks). In terms of the quality of life, exercise intervention showed a tendency to increase PHS and MHS, but this did not reach statistical significance. Taken together, these findings indicate that overweight/obese breast cancer survivors may benefit from exercise intervention for weight management.

Weight control is essential for patients with breast cancer, as overweight and obesity have been found to contribute to the recurrence and progression of breast cancer, negatively affecting its prognosis [39]. A high BMI is linked to higher risk and worse clinical outcomes in patients with breast cancer [40], while over half of the breast cancer survivors are overweight or obese [18, 41]. Exercise intervention has been shown to reduce BMI in patient with breast cancer [41]. Among breast cancer survivors, exercise significantly reduces body weight and waist circumference [33] and illustrated positive outcomes with BMI, lean mass, and muscle strength [42]. In the present study, we have extended these findings showing a favorable effect of exercise intervention on BMI among overweight/obese breast cancer survivors.

Given that BMI only reflects the relationship between height and weight and not the distribution of body fat, this study further demonstrated the favorable effect of exercise on body fat among overweight/obese breast cancer survivors. Body fat is positively correlated with the activation of the mTOR pathway, which is associated with tumor growth in breast cancer patients [43]. Fat body mass and lean body mass synergistically predict the risk of morphometric vertebral fractures in breast cancer patients who received aromatase inhibitors, a drug commonly used after chemotherapy that may lead to bone loss and elevated fracture risk [44, 45]. Lauby-Secretan et al. suggested that lower body fat may reduce the risk of various cancers [46]. As such, the synergistic results from this study, exercise reduces body fat, may provide practical guidance for the recovery plans for breast cancer survivors.

It is noteworthy that such exercise effects on these outcomes were moderated by the intervention durations. The current meta-analysis indicates the effects of exercise were better on BMI, fat mass, and bone mineral density at the relatively shorter program (12-week, 16-week) but not at long-term program (52-week); while the effects on lean mass were better in both short-term and long-term program. For example, Carayol et al. showed that adapted physical activity and diet intervention significant decreased BMI and fat mass at 26 weeks, but such effects did not persist afterward [47]. Juvet et al. indicated that exercise intervention could produce short-term improvements in physical functioning, and the time-dependent observations should be confirmed based on more studies [48]. The mechanism of different effects of intervention durations is not completely clear yet. Some researchers comment that the cessation of supervision and support may contribute to the difficulties of maintaining exercise in long-term exercise intervention [49]. The unique conditions of breast cancer survivors (e.g., breast cancer type, course of disease, treatment methods, and adverse reactions), may also interact with environmental influences to facilitate or hinder the weight management progress [50].

Another reason for the different effects of intervention durations might be the confounding effects of exercise intensities. Exercise intensity is a key factor in exercise intervention, which determines the safety and effectiveness of exercise interventions for patients. The ideal exercise effect cannot be achieved when exercise intensity does not reach the minimum threshold. In contrast, it may lead to overtraining and joint damage when the intensity exceeds the maximum threshold. The American College of Sports Medicine roundtable recommends moderate-intensity (> 30 min for > 3 times per week) aerobic exercise for at least 8–12 weeks for cancer survivors to obtain health-related outcomes, or similar benefits are also obtained by combining resistance exercise with aerobic exercise at least 2 times per week, using at least 2 sets of 8–15 repetitions at least 60% of one repetition maximum [51]. In this meta-analysis, exercise prescriptions differed among RCTs and some did not provide specific intensities. Given the limited studies and the inconsistent effect of exercise durations, we should be with cautious when treating the association between exercise and the change of body compositions for overweight/obese breast cancer survivors, and more research is needed before making a confirmed conclusion.

The potential benefits of exercise in improving quality of life of breast cancer survivors have been reported in studies [18, 52]. In a systematic review based on 26 RCTs, Hong et al. concluded that exercise intervention substantially improved the quality of life of breast cancer survivors, and the improved quality of life was associated with “time of session” [53]. Another two meta-analyses also found that exercise intervention improved the quality of life in breast cancer survivors, including social well-being, functional well-being, emotional well-being, and physical well-being [54], mental health and general health [42]. However, our meta-analysis indicated that exercise intervention tended to increase PHS and MHS of overweight/obese breast cancer survivors, but did not reach statistical significance. The differences on the number of included studies might partly explain such inconsistent conclusion as only two studies reporting the differences in PHS or MHS of overweight/obese breast cancer survivors between exercise and control groups were included. On the other hand, the quality of life of breast cancer survivors is heavily influenced by the treatment (e.g., selective estrogen receptor modulator), which may cause a series of physical and psychological impairment [55]. Sexual health can also be negatively impacted after breast cancer and therefore influence their quality of life [56]. Specific treatment modalities has been suggested to improve sexual health and quality of life collaboratively for breast cancer survivors [57]. Furthermore, exercise can improve sleep quality in breast cancer survivors [58], and therefore improve their overall quality of life [59]. Thus, understanding breast cancer survivors’ conditions and treatment plans is critical while prescribing exercise treatment.

This meta-analysis has several limitations. First, although there was low methodological heterogeneity among the included RCTs, most of the RCTs were conducted in the United States, which limited the extrapolation of the results. Second, meta-analysis indicated that exercise intervention could significantly decrease the BMI of breast cancer survivors in comparison with that of routine care. However, the study of Dieli-Conwright et al. [24] weighted as high as 52.6% among the four studies, and revealed the significant exercise intervention effect on BMI [WMD (95% CI) = -2.10 (-3.66, -0.54) kg/m^2^, P < 0.05], while there were no significant effects on BMI in the other studies. In the sensitivity analysis, the pooled results for BMI changed from significant to non-significant after eliminating the study of Dieli-Conwright et al. [24], indicating an unstable result. Third, the number of included RCTs was small, and significant heterogeneity among studies was observed for multiple outcome indexes. Therefore, more evidence from high-quality RCTs with larger sample sizes is needed to confirm the benefits of exercise interventions for overweight/obese breast cancer survivors.

## Conclusion

This meta-analysis indicated that exercise intervention could significantly decrease BMI and body fat of overweight/obese breast cancer survivors. Although there was no statistical significance, exercise intervention decreased body weight and fat mass, and increased lean mass and bone mineral density under different exercise intervention duration. A tendency of improved quality of life was also detected. These findings suggest the benefits of exercise interventions in overweight/obese breast cancer survivors, while more evidence is needed for a conclusive result.

### Electronic supplementary material

Below is the link to the electronic supplementary material.

Supplementary Material 1: Supplementary figure 1. Methodological quality evaluation for the included studies

Supplementary Material 2

Supplementary Material 3

## Data Availability

The datasets used and/or analysed during the current study are available from the corresponding author on reasonable request.
