# Correction: Exercise interventions on body composition and quality of life of overweight/obese breast cancer survivors: a meta-analysis

**DOI:** 10.1186/s12905-023-02661-0

**Published:** 2023-09-27

**Authors:** Hongchang Yang, Li Liu, Xiaoxia Zhang

**Affiliations:** 1 Physical Education Department, Hohai University, Nanjing, Jiangsu China; 2 Department of Rehabilitation, Brain Hospital Affiliated to Nanjing Medical University, No.264, Guangzhou Road, Gulou District, Nanjing, Jiangsu China; 3 Department of Kinesiology, Centenary College of Louisiana, 2911 Centenary Blvd, Shreveport, Louisiana, LA USA

Following publication of the original article [1], in this article the affiliation details for Authors Li Liu^2^ and Xiaoxia Zhang^3^ were incorrectly given as “^2^Department of Rehabilitation, Brain Hospital Affiliated to Nanjing Medical University, 2911 Centenary Blvd, Shreveport, Louisiana, LA, USA; ^3^Department of Kinesiology, Centenary College of Louisiana, No.264, Guangzhou Road, Gulou District, Nanjing, Jiangsu, China” but it should read as “^2^Department of Rehabilitation, Brain Hospital Affiliated to Nanjing Medical University, No.264, Guangzhou Road, Gulou District, Nanjing, Jiangsu, China; ^3^Department of Kinesiology, Centenary College of Louisiana, 2911 Centenary Blvd, Shreveport, Louisiana, LA, USA ”.

The original article has been corrected.
