# Supplementary figures and images for: Exercise interventions on body composition and quality of life of overweight/obese breast cancer survivors: a meta-analysis
Source: BMC Womens Health. 2023 Sep 12;23:484. doi: 10.1186/s12905-023-02627-2 (PMC10498647; doi:10.1186/s12905-023-02627-2)

**
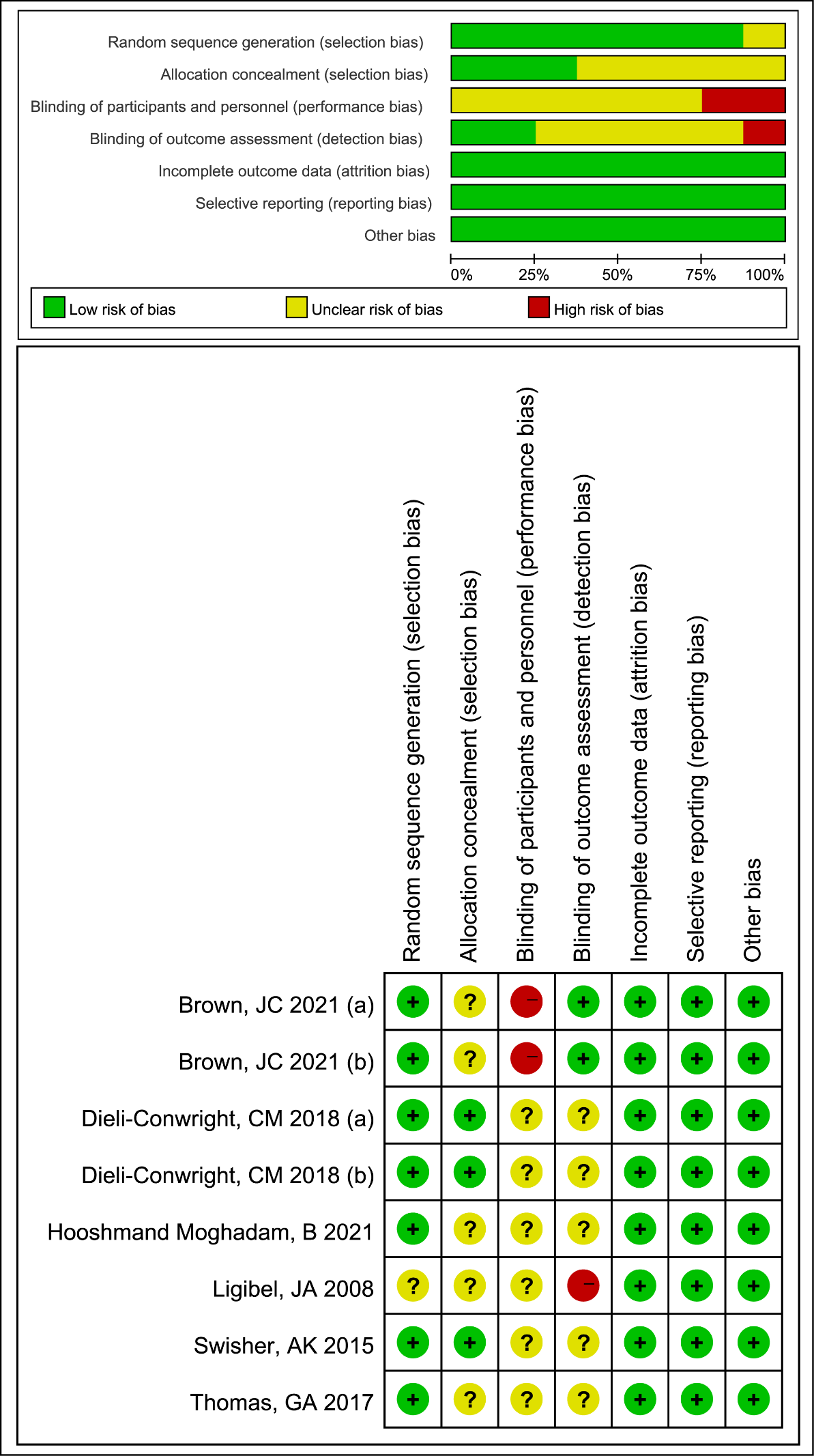
**

**Supplementary figure 1.** Methodological quality evaluation for the included studies

Supplement: Supplementary file 1 — Supplementary Material 1: Supplementary figure 1. Methodological quality evaluation for the included studies [file 12905_2023_2627_MOESM1_ESM.docx]
