# Supplementary material for: Exercise interventions on body composition and quality of life of overweight/obese breast cancer survivors: a meta-analysis
Source: BMC Womens Health. 2023 Sep 12;23:484. doi: 10.1186/s12905-023-02627-2 (PMC10498647; doi:10.1186/s12905-023-02627-2)
Supplement: Supplementary file 2 — Supplementary Material 2 [file 12905_2023_2627_MOESM2_ESM.docx]

**Supplementary table 1 The complete retrieval strategies for studies in PubMed, Embase, cochrane library databases**

| PubMed database | | |
| --- | --- | --- |
| Search | Query | Items found |
| #1 | ("overweight"[MeSH Terms] OR "overweight"[All Fields] OR "overweighted"[All Fields] OR "obeses"[All Fields] OR "obesity"[MeSH Terms] OR "obesity"[All Fields] OR "obese"[All Fields] OR "obesities"[All Fields] OR "obeses"[All Fields]) | 438723 |
| #2 | ("exercise"[MeSH Terms] OR "exercise"[tiab] OR "physical activity"[tiab] OR ("sports"[MeSH Terms] OR "sports"[tiab] OR "sport"[tiab] OR "sporting"[tiab]) OR ("training"[tiab] OR "education"[MeSH Terms] OR "train"[tiab] OR "trained"[tiab] OR "trainings"[tiab] OR "trains"[tiab]) OR ("exercises"[tiab] OR "exercise therapy"[MeSH Terms] OR "exercise therapy"[tiab] OR "exercised"[tiab] OR "exerciser"[tiab] OR "exercisers"[tiab] OR "exercising"[tiab])) | 1875565 |
| #3 | ("breast neoplasms"[MeSH Terms] OR "breast neoplasms"[tiab] OR "breast cancer"[tiab]) | 416486 |
| #4 | ((randomized controlled trial[pt] OR controlled clinical trial[pt] OR randomized[tiab] OR randomised[tiab] OR placebo[tiab] OR drug therapy[sh] OR randomly[tiab] OR trial[tiab] OR groups[tiab]) NOT (animals[mh] NOT humans[mh])) | 4767849 |
| #5 | #1 AND #2 AND #3 AND #4 | 394 |
| Embase database | | |
| Search | Query | Items found |
| #1 | ('overweight'/exp OR overweight OR obese OR 'obesity'/exp OR obesity) | 650644 |
| #2 | ('exercise'/exp OR 'exercise' OR 'physical activity'/exp OR 'physical activity' OR 'sports'/exp OR 'sports' OR 'sport'/exp OR 'sport' OR 'sporting' OR 'training'/exp OR 'training' OR 'train'/exp OR 'train' OR 'trainings' OR 'trains' OR 'exercises' OR 'exercising') | 1538740 |
| #3 | ('breast neoplasms'/exp OR 'breast neoplasms' OR 'breast cancer'/exp OR 'breast cancer') | 597661 |
| #4 | (crossover AND ('procedure'/exp OR procedure)) OR (double AND ('blind'/exp OR blind) AND ('procedure'/exp OR procedure)) OR (randomized AND controlled AND ('trial'/exp OR trial)) OR ('single blind' AND ('procedure'/exp OR procedure)) OR (random* OR factorial* OR crossover* OR (cross AND over*)) OR placebo* OR (doubl* AND adj AND blind*) OR (singl* AND adj AND blind*) OR assign* OR allocat* OR volunteer* | 2677475 |
| #5 | #1 AND #2 AND #3 AND #4 | 707 |
| Cochrane library | | |
| Search | Query | Items found |
| #1 | MeSH descriptor: [Overweight] explode all trees | 18878 |
| #2 | MeSH descriptor: [Obesity] explode all trees | 15762 |
| #3 | (overweight OR obese OR obesity):ti,ab,kw (Word variations have been searched) | 50815 |
| #4 | #1 OR #2 OR #3 | 50898 |
| #5 | MeSH descriptor: [Sports] explode all trees | 17130 |
| #6 | MeSH descriptor: [Exercise] explode all trees | 28350 |
| #7 | (exercise OR "physical activity" OR sports OR sport OR sporting OR training OR train OR trainings OR trains OR exercises OR exercising):ti,ab,kw (Word variations have been searched) | 216645 |
| #8 | #5 OR #6 OR #7 | 219061 |
| #9 | MeSH descriptor: [Breast Neoplasms] explode all trees | 14567 |
| #10 | ("breast neoplasms" OR "breast cancer"):ti,ab,kw (Word variations have been searched) | 39201 |
| #11 | #9 OR #10 | 39226 |
| #12 | #4 AND #8 AND #11 | 416 |
| #13 | #12 in Trials | 415 |
